# Orthogeriatric Fracture Syndrome: A Large-Scale Bibliometric Analysis of a Proposed Concept for Cross-Disciplinary Awareness and Coordinated Care

**DOI:** 10.3390/jcm15083105

**Published:** 2026-04-18

**Authors:** Alceu Bissoto, Heike Annette Bischoff-Ferrari, Karin Blum, Silvia Brunner, Michael Dietrich, Serge Ferrari, Stefan Goetz, Slavko Rogan, Anke Scheel-Sailer, Lisa Margret Koch, Johannes Dominik Bastian

**Affiliations:** 1 Department of Diabetes, Endocrinology, Nutritional Medicine and Metabolism, Inselspital, Bern University Hospital, University of Bern, 3010 Bern, Switzerland; 2 Department of Digital Medicine, University of Bern, 3012 Bern, Switzerland; 3 Diabetes Center Berne, 3010 Bern, Switzerland; 4 Department of Geriatrics, University of Basel, 4002 Basel, Switzerland; 5 Department of Aging Medicine, FELIX Platter Basel, University of Basel, 4002 Basel, Switzerland; 6 Fragility Fracture Network, 3010 Bern, Switzerland; 7 City Hospital Zurich, 8063 Zurich, Switzerland; 8 Division of Bone Diseases, Geneva University Hospital, 1211 Geneva, Switzerland; 9 St. Katharinental Clinic, Diessenhofen, Spital Thurgau AG, Thurmed AG, 8253 Diessenhofen, Switzerland; 10 Department of Health Professions, Bern University of Applied Sciences, 3008 Bern, Switzerland; 11 Centre for Rehabilitation and Sports Medicine, Inselspital, University Hospital Bern, 3010 Bern, Switzerland; 12 Department of Orthopaedic Surgery and Traumatology, Inselspital, Bern University Hospital, University of Bern, 3010 Bern, Switzerland

**Keywords:** orthogeriatric fracture syndrome, hip fracture, frailty, interdisciplinary, correlation, large-scale text mining

## Abstract

**Background/Objectives**: Older patients with fractures often present with a complex interplay of factors associated with frailty and functional decline. The emerging concept of Orthogeriatric Fracture Syndrome (OFS) aims to characterize these distinct relationships of pathologies and outcomes. Despite increasing recognition of OFS in clinical practice, due to the distributed nature of fragility factors across medical disciplines, it remains poorly defined in the literature. **Methods**: We used large-scale text mining of 26 million PubMed abstracts to quantify the occurrence and interrelationship of OFS-related concepts across all disciplines in biomedical research. **Results**: OFS terms were more prevalent in fragility fractures than in other fracture types, particularly osteoporosis (0.52 vs. 0.09, *p* < 0.05). In pairwise keyword correlation (Pearson φ), the correlations presented between OFS keywords are comparable to the ones in the more established metabolic syndrome (e.g., φ = 0.07 between stroke and hypertension, *p* < 0.05). For OFS, osteoporosis emerged as the central node linking OFS outcomes and pathologies, correlating with fragility fracture (φ = 0.176, *p* < 0.05) and sarcopenia (φ = 0.03, *p* < 0.05). Sarcopenia in turn correlated with gait (φ = 0.04, *p* < 0.05), malnutrition (φ = 0.05, *p* < 0.05), and frailty (φ = 0.032, *p* < 0.05). Old age keywords showed substantially higher association with OFS keywords (e.g., φ = 0.06 for elderl* and hip fracture, *p* < 0.05) than with metabolic syndrome terms (elderl* and insulin resistance, *p* > 0.05). **Conclusions**: Overall, the analysis showed statistically significant associations between keywords representing OFS outcomes, pathologies and old age. The combined occurrence of osteoporosis, sarcopenia, frailty and risk of falls may help conceptually identify older adults at risk and inform preventive measures. This large-scale bibliometric analysis supports OFS as a conceptually coherent, proposed theoretical framework for cross-disciplinary awareness and coordinated care, with a literature-level organizational pattern comparable to metabolic syndrome, however, pending prospective clinical validation. This study reframes fragility fractures as the endpoint of a broader, potentially modifiable risk constellation and underscores the need for further clinical and epidemiological validation.

## 1. Introduction

In medicine, a syndrome is defined as the coexistence of multiple characteristic clinical features or abnormalities that occur in a typical pattern and reflect a shared underlying pathophysiological background, without constituting a single disease entity [1,2]. Metabolic syndrome represents a well-established example of this construct, integrating central obesity, insulin resistance, dyslipidemia, and hypertension into a clinically silent yet prognostically relevant risk constellation that may proceed to adverse cardiovascular events such as stroke and heart attack [3,4]. Its recognition as a distinct clinical syndrome has substantially increased awareness among healthcare professionals and enabled interdisciplinary prevention strategies [4].

In contrast, the term fragility fracture semantically denotes a discrete and clearly delineated clinical event, typically triggered by low-energy trauma, and is therefore understood as the visible endpoint of an otherwise largely silent decline. Etymologically, the term syndrome (from the Greek σύνδρομος—“running together”) refers to a recognizable pattern of co-occurring conditions rather than an isolated manifestation. Building on this semantic-etymological distinction, we propose Orthogeriatric Fracture Syndrome (OFS) as a proposed conceptual framework describing the underlying syndromic process from which a fragility fracture emerges: a multidimensional constellation of osteoporosis, sarcopenia, malnutrition, gait impairment, elevated fall risk, frailty and cognitive impairment that progressively converges toward the orthogeriatric fracture event (e.g., hip fracture). Recognizing OFS as a formal syndrome therefore clarifies the semantic relationship between the event (fragility fracture) and the process (OFS) and may enhance interdisciplinary awareness and collaboration across primary, secondary, and tertiary fracture prevention.

The objective of this study was to determine whether OFS-related pathologies exhibit a structured and non-random pattern of interconnection within the biomedical literature. We hypothesized that these conditions co-occur in a characteristic manner comparable to metabolic syndrome. Using large-scale text mining of academic literature, we quantified the occurrence and interrelationship of OFS-related concepts across biomedical disciplines to examine whether the OFS shows conceptual coherence in the biomedical literature.

## 2. Materials and Methods

Our goal is to examine whether the proposed Orthogeriatric Fragility Syndrome (OFS) is reflected as a coherent concept across the biomedical literature. We propose using data analysis methods to search for relevant keywords in paper abstracts and then quantify the relationships between OFS-related keywords using statistical analysis techniques. We leveraged large-scale text mining of PubMed abstracts to (i) identify terms disproportionately enriched in “fragility-fracture” papers and (ii) quantify the strength of association between those terms.

### 2.1. Data

We used the 2026 version of the complete PubMed database for our analyses, which is made available yearly for bulk download. The dataset contains all approximately 40 M articles indexed by PubMed which were published until the end of 2025. We follow the same preprocessing of [5]. We eliminated records with missing abstracts, those outside the 250–4000 character range, and unfinished abstracts missing standard end-of-sentence punctuation (‘.’, ‘?’, or ‘!’). Finally, the cleaned dataset contained 26 M articles. It includes articles’ title, authors, year, abstract, and journal of publication. We preprocessed the data following the previous literature [5,6]. In addition, we used tools for two-dimensional visualizations of the entire landscape of biomedical research, which were also provided by [5,6]. All analyses were performed using Python 3.10.16 with the following key libraries: numpy 1.26.4, pandas 2.2.3, scipy 1.11.4, statsmodels 0.14.4, openTSNE 1.0.2, and matplotlib 3.10.1.

### 2.2. Keywords and Search Terms

To annotate our dataset appropriately for investigating OFS, we selected keywords based on four categories:

1. Outcomes and pathological characteristics specifically associated with OFS: hip fracture, femur fracture, spine fracture, fragility fracture, fracture, fragility (outcomes); and osteoporosis, sarcopenia, malnutrition, gait, fall, frail, cognitive impairment (pathologies).
2. Patient’s age-related characteristics: older adult, elderl*, geriatr*. The “*”at the end of some words indicates a prefix match (e.g., “geriatr*” matches geriatric, geriatrics, and geriatrician).
3. Control keywords negatively associated with fragility fractures to act as comparative baselines: sport, athlet*, trauma, young, high-energy.
4. Outcomes and pathological characteristics of the well-established metabolic syndrome, serving as a reference syndrome for validation purposes. Metabolic syndrome was chosen as a baseline for comparison due to its well-characterized clinical profile and established presence in the literature: stroke, infarction, heart failure, heart attack (outcomes); and insulin resistance, obesity, hypertension, diabetes (pathologies).

Keywords were suggested by an experienced clinical expert specialized in orthopedic trauma surgery and orthogeriatric medicine and then adjusted by an interprofessional and interdisciplinary panel of scholars in the field of orthogeriatric treatment (Board of Fragility Fracture Network Switzerland, compare co-authors). The final keyword set was defined through an iterative interdisciplinary expert discussion. Selection was based on perceived clinical relevance, conceptual coherence, and consistency with existing guideline-informed constructs. As no formal Delphi procedure or predefined numerical agreement threshold was used, the consensus process should be understood as qualitative rather than quantitative.

The abstracts in the PubMed dataset were then queried for the occurrence of the selected keyword, resulting in a binary matrix denoting the presence or absence of each keyword in each abstract.

### 2.3. Contrasting Fragility Fractures with Trauma Fractures

Next, we analyzed the data to uncover the keywords associated with fragility fractures. For that, we first created a filtered version of the dataset containing only articles mentioning “fragility” and “fracture” in their abstracts. To isolate the effects on fragility fractures instead of other traumatic fractures, we created another subset of articles mentioning “fracture” without “fragility”. We used this as a comparison to identify keywords that are not specific to fragility fractures.

We then proceeded to compare the frequency of the annotated keywords between these two sets. Keywords that are significantly more frequent in the “fragility fracture” set are indicative of positive associations with OFS, whereas keywords more frequent in the “other fractures” set are negatively associated or unrelated to OFS. For statistical significance, we ran a two-proportion z-test, Bonferroni-adjusted.

### 2.4. Statistical Analysis to Quantify Relationships Between Keywords

With annotated keywords, we systematically examined their presence in each abstract, marking occurrences as binary indicators (present or absent). We subsequently constructed a co-occurrence matrix, where each entry (i,j) quantifies the frequency that keywords i and j are co-existing across abstracts.

Every possible pair of keywords thus formed a 2 × 2 table showing how often both were present, only one was present, or neither were present. The strength and direction of each association were summarized using the φ-coefficient, which is equivalent to the Pearson correlation for two binary variables and ranges from −1 to +1. Positive correlations indicate pairs of keywords consistently appearing together in abstracts, suggesting a potential meaningful association within the syndrome. To contextualize and validate our findings, we employ metabolic syndrome-related keywords as baselines to benchmark correlations specific to OFS. To test whether each pair of keywords was associated, we used the Pearson χ^2^ test when all four cells in the table contained at least five documents; otherwise, we used Fisher’s exact test, which is valid for small counts. Because many keyword pairs were analyzed, we adjusted the raw *p*-values using the Holm false discovery rate (FDR) procedure to control for multiple comparisons. Results with q < 0.05 (indicating an expected false positive rate below 5%) were considered statistically significant.

### 2.5. Visualization of OFS Literature in the Context of Published Biomedical Research

For qualitative inspection, we visualized literature related to OFS in the context of the whole of biomedical research. Here, we used the tools provided by [5], who used machine learning to represent each abstract as a point in two dimensions. Ref. [5] used natural language processing techniques to represent each paper abstract as a high-dimensional feature vector and then applied t-distributed stochastic neighbor embedding (t-sne) [7] to embed these feature vectors in two-dimensional space to facilitate visualization and interpretation. The representations were learned such that similar abstracts occupied similar areas in two-dimensional space, and unrelated abstracts were further apart.

## 3. Results

### 3.1. Dataset Summary Statistics

**Figure 1:**
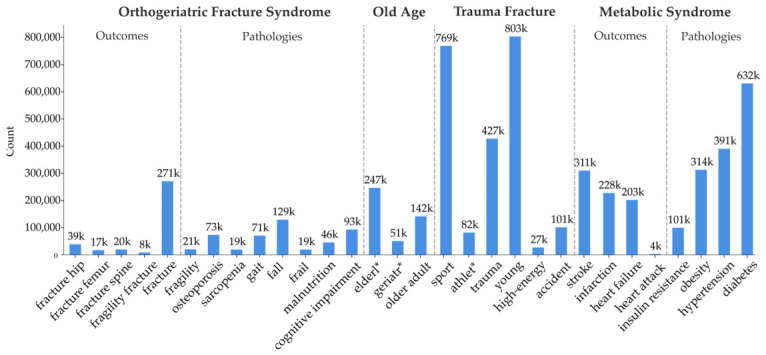
Number of article abstracts (vertical axis) which contained our keywords of interest (horizontal axis). The keywords were grouped by concepts which we hypothesize to be OFS-related, concepts related to old age, concepts related to trauma fractures rather than fragility fracturs, and concepts related to metabolic syndrome as a comparison.

We analyzed a total of 26 million paper titles and abstracts, with keywords assigned based on their presence in the abstract. Figure 1 presents the occurrence counts grouped into concepts related to Orthogeriatric Fracture Syndrome (OFS), with metabolic syndrome included as a comparator. The term “fracture” appeared in approximately 271,000 papers, forming the foundation of our analysis, which primarily examines associations between the fracture-related literature and keywords pertaining to age, bone-specific conditions, and fracture etiology. OFS outcome keywords represent subsets of this broader fracture category, encompassing hip, femur, and spine fractures, as well as fragility fracture in general. Trauma and old-age keywords exhibit high frequency as they extend beyond fracture-specific contexts (e.g., sports, athletes, accidents), yet remain important for subsequent analysis of their association with the fractures under investigation.

**Figure 2:**
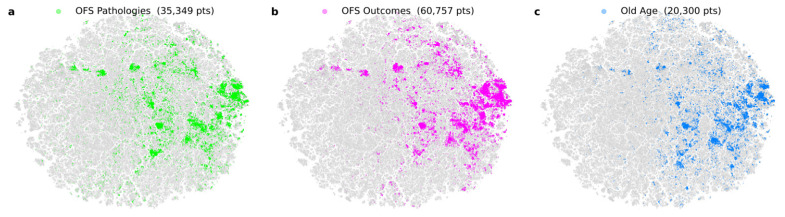
Grouped OFS-related keywords and its disposition in the landscape of biomedical research. (**a**) OFS Pathologies, (**b**) OFS Outcomes, and (**c**) Old-Age-related keywords consistently overlap, evidencing there is a consistent relationship between them and a potential characterization of a syndrome.

To assess semantic relationships among keywords, we compared the positions of abstract embeddings pre-computed by [5,6] where they used data from 2024 (Figure 2). Keywords were color-coded by group, revealing substantial overlap among OFS Pathologies (Figure 2a, green), OFS Outcomes (Figure 2b, magenta), and Old-Age-related terms (Figure 2c, blue). This indicates that abstracts associated with different keywords nonetheless share similar content, providing supporting evidence for the conceptual association among OFS-related terms.

**Figure 3:**
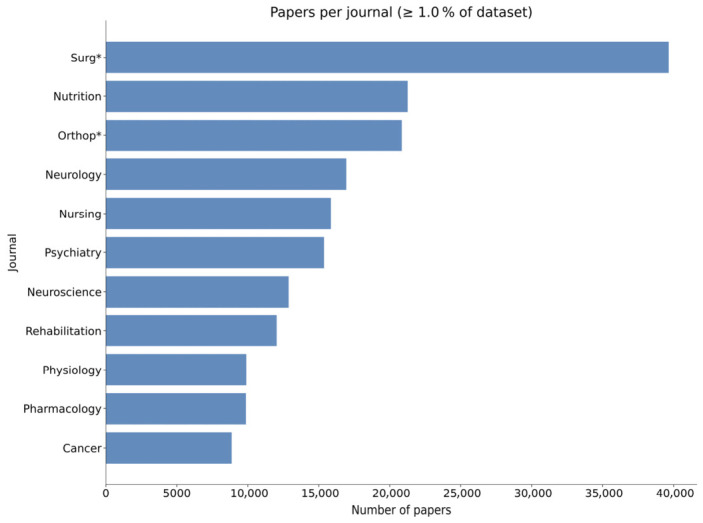
The research areas where OFS keywords appear most. The main areas represented are “Surgery”, “Nutrition”, “Orthopedics” and “Neurology”.

Figure 3 presents the distribution of selected keywords across medical disciplines, using the target audience of the journal as a proxy for the medical discipline. As expected, orthopedics and surgery journals contained the highest concentration of OFS-associated papers. This distribution serves as validation that our analysis captures literature from relevant domains rather than unrelated fields.

### 3.2. Fragility Fractures Are Specifically Linked to OFS Keywords

**Figure 4:**
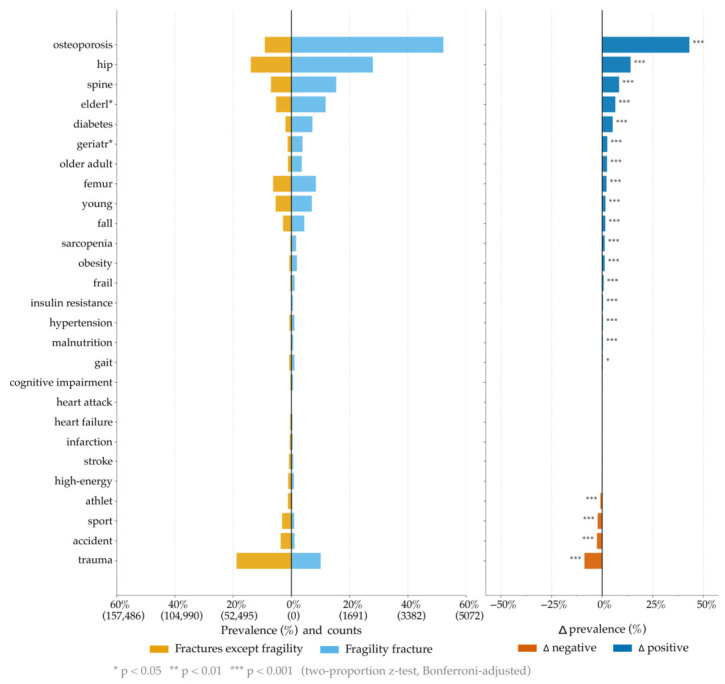
Left: This plot contrasts the presence of keywords (y-axis) in two sets: (background—yellow bars) “fracture without fragility”; (query—light blue bars) “fragility fracture”. Percentages and absolute sample counts in the x-axis. Right: The difference between the bars on the left plot. It highlights the keywords that are specifically connected to fragility fracture (opposed to fracture without fragility).

Next, we contrasted fragility fractures with non-fragility fractures (methodology explained in Section 2.3), and verified OFS keywords carried significantly stronger associations with fragility fractures (Figure 4). For example, the term “osteoporosis” was prevalent in >50% of abstracts about fragility fractures, compared to approximately 10% in abstracts on non-fragility fractures (Figure 4: left, top row). This constituted the largest, and statistically significant (*p* < 0.001), difference in prevalence (Figure 4: right) of approx. 40%. The analyzed keywords were ranked by prevalence difference (Figure 4: right), revealing additional strong positive associations between fragility fractures and the keywords “hip”, “spine”, “elderl*”. In contrast, the keywords “athlet*”, “sport”, “accident”, and “trauma” were significantly (*p* < 0.001) negatively associated with abstracts on fragility fractures. In summary, abstracts on fragility fractures were consistently positively associated with OFS keywors and negatively associated with trauma-related keywords.

### 3.3. OFS Outcomes Are Correlated with Older Age and Osteoporosis and OFS Pathologies Are Interrelated

**Figure 5:**
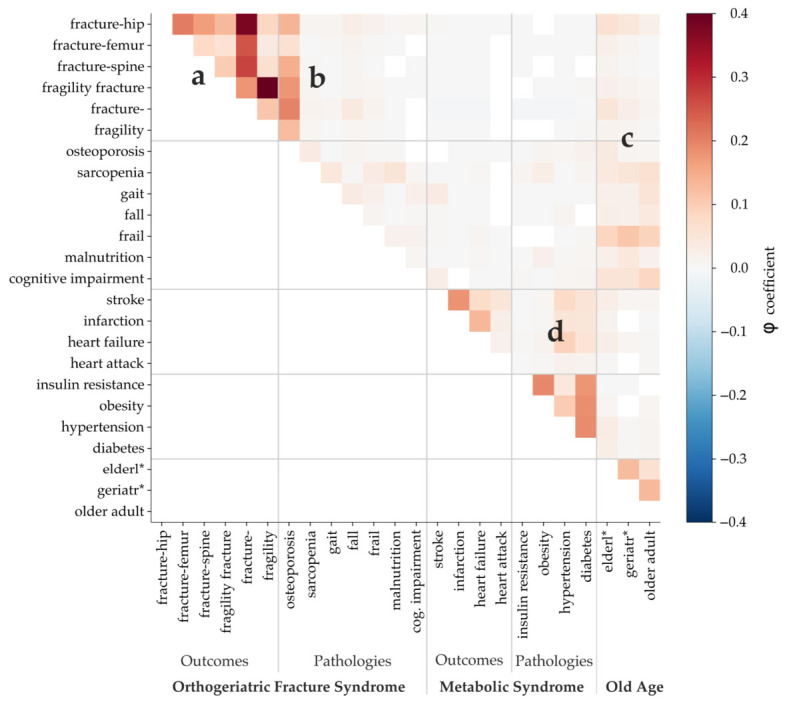
Pearson correlations for co-occurring keywords. (a) OFS outcomes are strongly correlated. Locations such as hip, femur and spine follow the same patterns of fragility fractures. (b) Osteoporosis presents high correlations with all the OFS outcomes. Moreover, correlations among OFS pathologies correlations are significant. (c) Old age keywords are correlated with OFS outcomes and pathologies, but not with metabolic syndrome keywords. (d) Correlations between metabolic syndrome’s pathologies and outcomes are comparable to OFS.

Finally, we calculated correlations for keywords co-occurring in article abstracts (methodology in Section 2.4). We expected OFS-related terms to present high correlations among themselves and low correlations with other terms, such as metabolic syndrome keywords, which we included for comparison. In Figure 5, we labeled regions of the heatmap to guide the reader through the most important patterns. All non-blank cells represent statistically significant correlations (*p* < 0.05).

In panel 5a, correlations among OFS outcomes (e.g., fracture-hip, fragility fracture) are considerably high. “Fragility fracture” appears associated especially with “fracture-hip”. In terms of OFS pathologies (panel 5b), “osteoporosis,” a key OFS pathology, shows high correlations with all OFS outcomes. Osteoporosis also correlates with “sarcopenia,” which in turn correlates with “gait,” and “frail”, which also correlates to “fall” and “cognitive impairment”. This chain of conditions reflects ortogeriatric syndrome and the associated pathologies.

Old age keywords show high correlations with OFS pathologies and outcome keywords (panel 5c), specifically with “fracture-hip”and “frail”, showing that this is also a key part to characterize OFS. Metabolic syndrome keywords show much milder correlations with old age.

### 3.4. OFS Present Comparable Correlations with Metabolic Syndrome Keywords

Panel 5d shows metabolic syndrome correlations as a contrast. The intensity of correlations within this cluster is slightly higher, as metabolic syndrome is a well-established and extensively studied condition. However, metabolic syndrome keywords do not show similar levels of correlation with old age as OFS does, nor do they show any correlations with OFS keywords. This distinction demonstrates that OFS correlations are not merely noise.

## 4. Discussion and Conclusions

This study supports the hypothesis that orthogeriatric fracture-related pathologies form a structured and non-random pattern of interconnection, conceptually similar to metabolic syndrome at the literature level. The large-scale analysis of 26 million publications demonstrates consistent co-occurrence and intercorrelations among OFS-related pathologies, outcomes, and age-associated terms, supporting the notion of a coherent conceptual risk constellation in the biomedical literature. Fragility fractures therefore appear, at least conceptually, not as isolated events but as the endpoint of a broader, often silent syndromic process that may be identified early and targeted through preventive interventions.

The concept of a syndrome has proven particularly valuable in medicine when multiple clinically silent abnormalities interact in a characteristic pattern and culminate in severe adverse outcomes. Metabolic syndrome represents a paradigmatic example of this approach, integrating metabolic and cardiovascular risk factors into a coherent risk constellation that precedes type 2 diabetes mellitus and major cardiovascular events [8]. Importantly, its formalization as a distinct clinical syndrome enabled early recognition, interdisciplinary awareness, and preventive intervention long before irreversible end-organ damage occurs. In contrast, fragility fractures in older adults are still predominantly approached as isolated events, despite extensive evidence on individual risk factors and their frequent coexistence. Importantly, the proposed OFS framework integrates several established but often separately studied domains of orthogeriatric risk, including osteoporosis, sarcopenia, frailty, falls, nutrition, mobility, and cognition.

Using large-scale text mining of 26 million PubMed abstracts, the present study provides evidence supporting the conceptual coherence of the proposed Orthogeriatric Fracture Syndrome (OFS) within the biomedical literature. Publications addressing fragility fractures showed a substantial overlap with keywords representing core OFS-related pathologies, including osteoporosis, sarcopenia, gait and balance impairment, fall risk, and frailty. Importantly, these concepts demonstrated a structured and non-random pattern of interconnection: OFS-related pathologies were significantly interlinked with one another, fracture-related outcomes were interlinked, and underlying pathologies were strongly associated with adverse outcomes. Notably, the observed relational structure closely resembled that of the metabolic syndrome, which served as a well-established exemplar syndrome. Together, these findings support the hypothesis that OFS represents a distinct and clinically meaningful conceptual risk constellation rather than a collection of isolated conditions.

From a pathophysiological perspective, this structured interconnection is highly plausible. Reduced bone strength due to osteoporosis compromises skeletal integrity, while sarcopenia reflects not only impaired muscle strength and diminished protective responses, but also broader reductions in functional capacity, physiological resilience, and metabolic regulation. Consistent with the close interrelationship between muscle loss and bone fragility, concurrent gait and balance impairment further increase instability, whereas elevated fall risk acts as the proximal trigger for fragility fractures [9,10,11]. Superimposed frailty reflects diminished physiological reserve and reduced resilience to stressors [12,13]. These impairments frequently coexist, interact synergistically, and remain clinically silent for prolonged periods [14]. Consequently, a minor mechanical insult may precipitate a major fragility fracture, which often represents the first overt manifestation of an advanced underlying syndrome. In this regard, fragility fractures may be conceptualized as the orthogeriatric analog of myocardial infarction or stroke in the metabolic syndrome.

A defining strength of metabolic syndrome is its operationalization through pragmatic diagnostic criteria. Although not a disease entity, it is commonly diagnosed when at least three of five components are present, including central obesity, insulin resistance or elevated fasting glucose, arterial hypertension, hypertriglyceridemia, and reduced high-density lipoprotein cholesterol [3]. This criteria-based approach transformed a diffuse risk profile into a clinically actionable framework and substantially improved prevention strategies [4]. Analogously, existing clinical knowledge and guidelines, informed by the present findings, provide a rationale to propose exploratory diagnostic criteria for OFS. Based on the existing clinical knowledge, guideline-based constructs, and the observed interconnections, OFS may be characterized by abnormalities across key domains, including impaired bone strength, reduced muscle mass or strength, gait stability and mobility, increased fall risk, and frailty as a marker of reduced physiological reserve. As a theoretical framework based on existing clinical guidelines, a pragmatic approach—analogous to the metabolic syndrome—could define OFS by the presence of impairments in at least four out of the following seven domains:

- The **bone strength domain** is defined by osteoporosis or elevated fracture risk, operationalized by a DXA T-score ≤ −2.5 at the hip or spine, a prior low-energy fragility fracture after the age of 50 years, or increased fracture probability based on validated risk assessment tools [15,16,17,18].
- The **muscle function domain** reflects sarcopenia and is operationalized by reduced muscle strength (e.g., handgrip strength below sex-specific cut-offs), low appendicular lean mass adjusted for height, or a diagnosis according to established consensus definitions [19,20,21].
- The **nutritional domain** reflects malnutrition and is operationalized according to the Global Leadership Initiative on Malnutrition (GLIM) criteria, requiring at least one phenotypic criterion (non-volitional weight loss, low body mass index, or reduced muscle mass) and one etiologic criterion (reduced food intake or assimilation, or disease burden/inflammation) [22].
- The **mobility domain** captures gait stability and mobility, defined by reduced gait speed, prolonged Timed Up and Go performance, objective balance deficits, or dependency on walking aids [23,24,25].
- The **fall risk domain** includes a history of one or more falls within the preceding 12 months, recurrent falls, fear of falling with activity restriction, or the presence of intrinsic or extrinsic risk factors such as polypharmacy or sensory impairment [26,27].
- The **physiological reserve domain** is represented by frailty, operationalized using validated instruments such as the frailty phenotype, the Clinical Frailty Scale, or deficit accumulation models [28,29,30,31,32].
- The **cognitive domain** reflects cognitive impairment and may be operationalized by a documented diagnosis of mild cognitive impairment or dementia, or by objective impairment on validated cognitive testing. In practice, brief screening instruments such as the Montreal Cognitive Assessment may be used as case-finding tools, followed by clinical confirmation [33,34,35,36].

Together, these domains describe a clinically silent yet synergistic risk constellation in which impairments in skeletal integrity, muscle function, mobility, and physiological reserve may interact to precipitate major fragility fractures. This proposed diagnostic framework is exploratory and intended to facilitate early identification and interdisciplinary prevention. It should be understood as a theoretical framework based on existing clinical guidelines rather than a direct finding of the present text-mining study. Its clinical validity and predictive performance require confirmation in prospective clinical and epidemiological studies. Importantly, such criteria should be regarded as hypothesis-generating and require validation in prospective clinical studies.

Several strengths and limitations of this study warrant consideration. A major strength lies in the comprehensive, data-driven approach, leveraging the full breadth of the biomedical literature across disciplines. Large-scale text mining enables the identification of non-random conceptual patterns that are not readily apparent in traditional single-disease or specialty-focused research. However, several limitations must be acknowledged. First, the analysis relied on automated text mining without manual content validation, allowing for potential misclassification or inclusion of publications not explicitly addressing fragility fractures. Second, while many keyword co-occurrences reached statistical significance, the associated φ-coefficients were modest in magnitude. However, in a corpus of 26 million abstracts, even a modest φ-coefficient reflects stable and reproducible co-occurrence patterns. Given the conservative Holm correction, significant associations reported here represent a lower bound on detectable co-occurrence patterns. Importantly, co-occurrence of terms within the literature reflects research patterns rather than direct causal relationships or clinical effect sizes. Accordingly, observed literature associations, while compelling, do not substitute patient-level clinical validation and describe the literature landscape of the concept rather than the clinical syndrome itself; the findings should be interpreted as hypothesis-generating and require validation in clinical, epidemiological, and interventional studies. Third, the present analysis may underestimate the complexity of aging, as it did not account for diseases beyond those selected for inclusion. Nevertheless, the combined occurrence of osteoporosis, sarcopenia, frailty and risk of falls may help conceptually identify older adults at risk and inform preventive measures. Lastly, a limitation of our search strategy is that, although we included the broader term fragility fracture, we did not explicitly list distal radius or proximal humerus fractures, which may have reduced sensitivity for studies specifically addressing upper-extremity fragility fractures in older adults. However, this was intentional, as our review focused on a geriatric population, in whom hip and vertebral fractures better reflect the fracture burden associated with frailty, disability, and loss of independence than upper-extremity fractures.

Despite these limitations, the clinical implications of recognizing OFS as a unifying conceptual framework are substantial. Establishing OFS as a distinct conceptual entity may increase awareness among healthcare professionals and promote interdisciplinary and interprofessional collaboration. The OFS may help to identify patients who would benefit from effective prevention and management of OFS, which requires coordinated care involving orthogeriatric teams, nutrition specialists, endocrinologists, rehabilitation experts, and clinicians addressing comorbid conditions. Orthogeriatric co-management (CoM) and fracture liaison services (FLS) are established and recommended models of care for older adults sustaining fragility fractures [37,38,39,40,41], with the overarching aims of reducing subsequent fracture risk, preventing functional decline, and preserving independence. Accordingly, recognizing this “OFS framework” as a distinct syndrome-like concept—analogous in its organizational logic to the metabolic syndrome—could improve cross-disciplinary awareness, facilitate more coordinated care pathways, and ultimately support the development of reimbursement frameworks.

In conclusion, this large-scale bibliometric analysis supports the concept of Orthogeriatric Fracture Syndrome as a coherent and structured constellation of interrelated pathologies and outcomes, analogous in its organizational pattern to metabolic syndrome. By confirming the interconnections among OFS-related concepts in the biomedical literature, this study provides a foundation for reframing fragility fractures as the endpoint of a broader, potentially modifiable risk constellation. Further clinical and epidemiological studies are warranted to validate OFS, refine its proposed theoretical diagnostic criteria, identify the most informative combinations of OFS domains and evaluate targeted preventive strategies in orthogeriatric care.

## Data Availability

The data used in this study are available at https://pubmed.ncbi.nlm.nih.gov/download/. All data processing and analysis code is publicly accessible at https://github.com/mlm-lab-research/ofs_pubmed. The preprocessing pipeline builds upon the repository https://github.com/berenslab/pubmed-landscape.
